# Direct measurement and correction of both megavoltage and kilovoltage scattered x‐rays for orthogonal kilovoltage imaging subsystems with dual flat panel detectors

**DOI:** 10.1002/acm2.12986

**Published:** 2020-07-25

**Authors:** Hiraku Iramina, Mitsuhiro Nakamura, Takashi Mizowaki

**Affiliations:** ^1^ Department of Radiation Oncology and Image‐applied Therapy Kyoto University Hospital Kyoto Japan; ^2^ Division of Medical Physics Department of Information Technology and Medical Engineering Faculty of Human Health Science Graduate School of Medicine Kyoto University Kyoto Japan; ^3^ Department of Radiation Oncology and Image‐applied Therapy Graduate School of Medicine Kyoto University Kyoto Japan

**Keywords:** dynamic tumor tracking, MV‐ and kV‐scatter, orthogonal imaging, radiotherapy, Vero4DRT

## Abstract

**Purpose:**

To measure the scattered x‐rays of megavoltage (MV) and kilovoltage (kV) beams (MV scatter and kV scatter, respectively) on the orthogonal kV imaging subsystems of Vero4DRT.

**Methods:**

Images containing MV‐ and kV‐scatter from another source only (i.e., MV‐ and kV‐scatter maps) were acquired for each investigated flat panel detector. The reference scatterer was a water‐equivalent cuboid phantom. The maps were acquired by changing one of the following parameters from the reference conditions while keeping the others fixed: field size: 10.0 × 10.0 cm^2^; dose rate: 400 MU/min; gantry and ring angles: 0°; kV collimator aperture size at isocenter: 10.0 × 10.0 cm^2^: tube voltage: 110 kV; and exposure: 0.8 mAs. The average pixel values of MV‐ and kV‐scatter (i.e., the MV‐ and kV‐scatter values) at the center of each map were calculated and normalized to the MV‐scatter value under the reference conditions (MV‐ and kV‐scatter value factor, respectively). In addition, an MV‐ and kV‐scatter correction experiment with intensity‐modulated beams was performed using a phantom with four gold markers (GMs). The ratios between the intensities of the GMs and those of their surroundings were calculated.

**Results:**

The measurements showed a strong dependency of the MV‐scatter on the field size and dose rate. The maximum MV‐scatter value factors were 2.0 at a field size of 15.0 × 15.0 cm^2^ and 2.5 at a dose rate of 500 MU/min. The maximum kV‐scatter value was 0.48 with a fully open kV collimator aperture. In the phantom experiment, the intensity ratios of kV images with MV‐ and kV‐scatter were decreased from the reference ones. After correction of kV‐scatter only, MV‐scatter only, and both MV‐ and kV‐scatter, the intensity ratios gradually improved.

**Conclusions:**

MV‐ and kV‐scatter could be corrected by subtracting the scatter maps from the projections, and the correction improved the intensity ratios of the GMs.

## 1 INTRODUCTION

In modern radiotherapy, high‐precision beam delivery has attracted considerable attention.^1^ Furthermore, several techniques for tracking tumors while considering patient‐specific respiratory motion have been developed, such as multileaf collimator (MLC) tracking,^2^ couch tracking,^3^, ^4^ real‐time tumor tracking,^5^ and dynamic tumor tracking (DTT) with a gimbaled x‐ray head.^6^

At our institution, infrared reflective (IR) marker‐based DTT has been applied to treat lung, liver, and pancreatic cancers using Vero4DRT (Mitsubishi Heavy Industries, Ltd., Hiroshima, Japan, and BrainLAB AG, Feldkirchen, Germany).^7^, ^8^, ^9^, ^10^ Vero4DRT has an O‐ring‐shaped gantry and two orthogonal kilovoltage (kV) imaging subsystems mounted ±45° from the megavoltage (MV) beam (Sources 1 and 2 and flat panel detectors [FPDs] 1 and 2).^11^, ^12^ In addition, an IR camera is mounted on the treatment room ceiling.

During beam irradiation, the target is tracked in real time by IR markers at four or five positions and a preconstructed correlational model between the IR marker positions and the three‐dimensional (3D) positions of the target, which are indicated by radiopaque markers.^13^, ^14^, ^15^ The predicted 3D target position is the average of the 3D target positions calculated from the IR markers. This approach also involves a four‐dimensional (4D) model, which is a quadratic polynomial equation. Two to four gold sphere markers (Olympus, Tokyo, Japan) and one flexible linear marker Visicoil (IBA dosimetry, Louvain‐la‐neuve, Belgium) are used for lung cancer and liver and pancreatic cancer, respectively.^7^, ^8^, ^9^, ^10^

Just before the first beam irradiation on each treatment day, the IR markers are monitored for 20 s, while the IR camera and radiopaque markers are detected by calculating the ratio between the intensity of the radiopaque marker and that of its surroundings in two orthogonal kV images simultaneously.^16^ The detected two‐dimensional (2D) radiopaque marker positions are converted into 3D positions using predefined camera parameters.^17^ Then, a 4D model is constructed by fitting datasets representing the IR marker and radiopaque marker positions into the equation.

At the time of beam irradiation, the 3D position of the marker is predicted by the IR marker position with the 4D model after 25 ms. Then, the gimbaled x‐ray head swings to the predicted position.^18^ During beam irradiation, the radiopaque markers are also detected on two orthogonal kV images in 1 s intervals. Both kV subsystems are always turned on during DTT treatment. The detected 2D positions are converted into 3D positions, and the differences between the converted and predicted 3D positions are calculated, using those positions visualized based on the concurrent kV images. If the difference exceeds a tolerance depending on the breathing pattern of the patient, Vero4DRT interrupts the MV beam irradiation automatically. In addition, the converted 3D radiopaque marker data can be used to rebuild a 4D model if necessary on the treatment day.

**Fig. 1:**
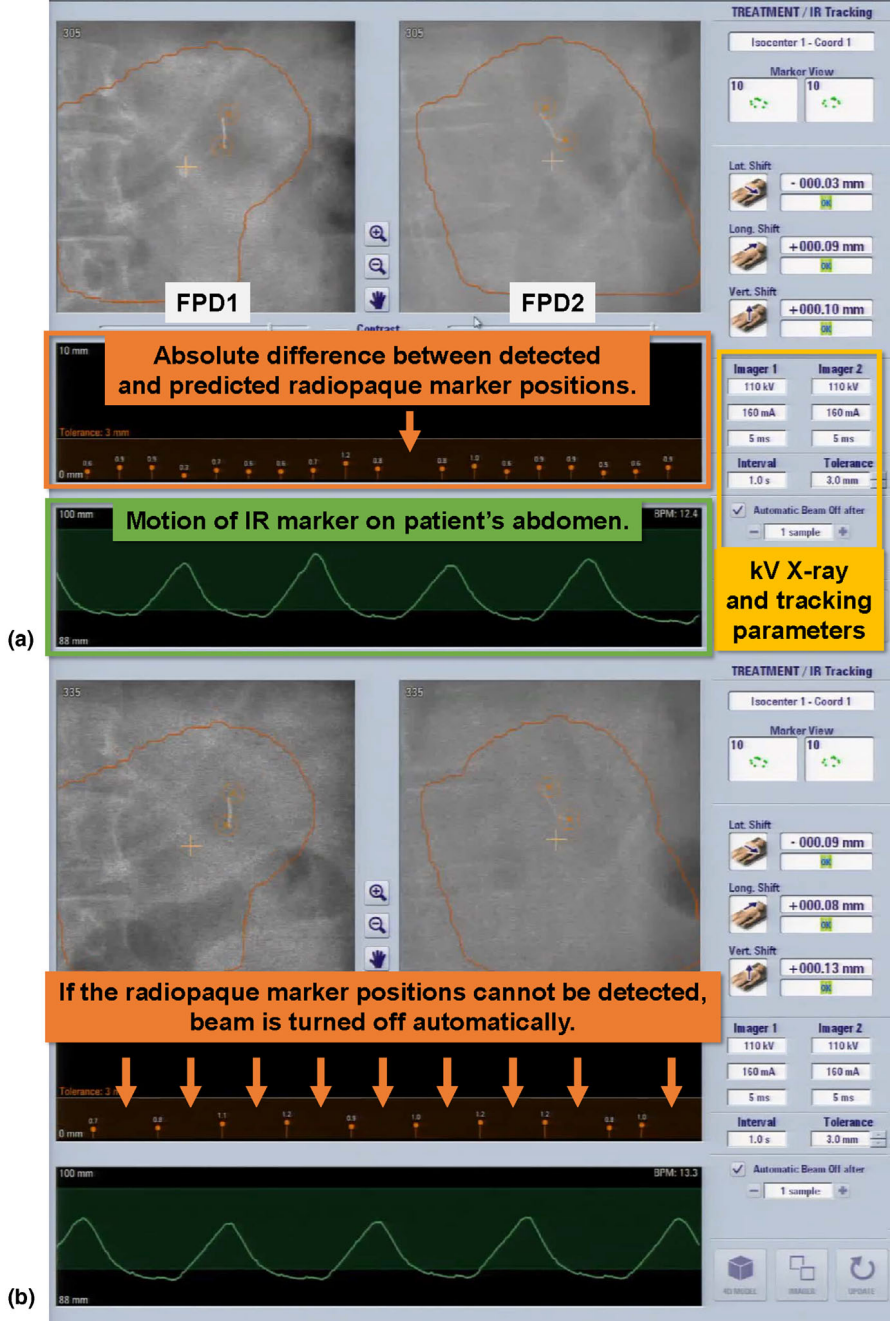
Example of treatment console during dynamic tumor tracking treatment using Vero4DRT. The projection images from flat panel detectors1 (FPD1) and FPD2, kV x‐ray and tracking parameters, infrared reflective marker motion, and absolute difference between the detected and predicted radiopaque marker positions are displayed. (a) If the radiopaque marker is detected without problems, the absolute difference can be calculated and shown. (b) If not, the absolute difference cannot be calculated and shown on the console (orange arrows). In addition, the contrast of the radiopaque marker is degraded by noise, especially in the image from FPD2.

As mentioned above, IR marker‐based DTT treatment requires concurrent kV imaging during MV beam irradiation. The concurrent kV images consist of primary and scattered kV x rays in addition to scattered x rays from the MV beam (MV‐scatter) and from the kV beam irradiated by the other kV source (kV‐scatter), which are scattered by the body of the patient. Thereby, the image contrast of the concurrent kV images is degraded by the MV‐ and kV‐scatter. Image contrast degradation can cause detection errors or lack of detection of the radiopaque marker, which leads to failure of the auto beam‐off system and online rebuilding of the 4D model. Figure 1 shows an example of the treatment console for DTT treatment in Vero4DRT. In Fig. 1(b), the concurrent kV images are degraded by MV‐ and kV‐scatter. Thereby, the absolute difference between the detected and predicted radiopaque marker positions was not calculated, and MV‐ and kV‐scatter in the Vero4DRT system have not been well investigated. Thus, the objectives of this study were to measure and quantify the MV‐ and kV‐scatter of two orthogonal kV imaging subsystems directly under various MV and kV beam parameters and to demonstrate MV‐ and kV‐scatter correction to improve radiopaque marker detection in a phantom study with intensity‐modulated beam irradiation

## 2 MATERIALS AND METHODS

### 2.A. Vero4DRT specifications

The gantry of Vero4DRT can rotate ±185° around the lengthwise axis of the patient couch (gantry rotation) and ±60° around the vertical axis (ring rotation). The nominal MV beam energy is 6 MV, and the maximum dose rate is 500 MU/min. The maximum field size (X × Y) is 15.0 × 15.0 cm^2^. The MV beam is collimated to patient‐specific field sizes by only MLCs with widths of 0.5 cm at the isocenter and heights and lengths of 11.0 and 26.0 cm, respectively. The source‐to‐axis distance is 100.0 cm. An amorphous Si electric portal imaging device (EPID) is mounted on the distal side of the MV beam.

**Fig. 2:**
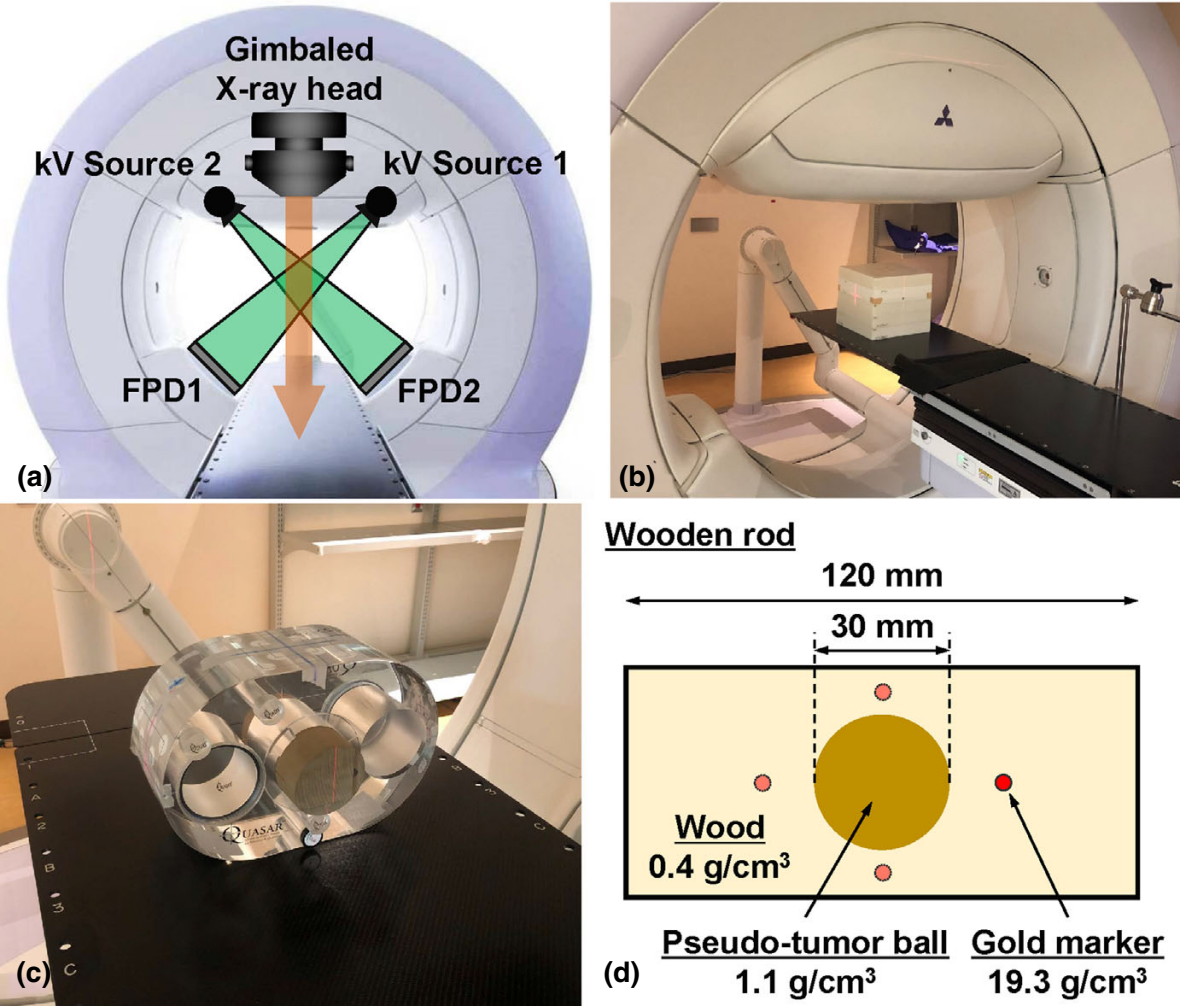
(a) Frontal view of Vero4DRT. (b) Experimental setup for this study. (c) Setup of the QUASAR phantom. (d) Schematic drawing of the wooden rod.

kV sources 1 and 2 are located at 45° and 315° in the orthogonal kV imaging subsystems, and FPDs 1 and 2 of the systems are located at 225° and 135°, respectively [Fig. 2(a)]. The kV sources and FPDs are located proximally and distally to the MV beam, respectively. The maximum kV source voltage of the orthogonal kV imaging subsystems is 125 kV, and the generated kV X ray can be manually collimated to arbitrary square or rectangular aperture sizes. The FPD for the subsystem is a PaxScan 4030CB FPD (Varian Medical Systems, Palo Alto, CA, USA) with an active imaging area and pixel matrix (transverse × longitudinal) of 39.7 × 29.8 cm^2^ and 1,024 × 768 pixels, respectively. The source‐to‐axis and isocenter‐to‐detector distances are fixed at 100.0 and 86.7 cm, respectively.

### 2.B. MV‐ and kV‐scatter measurements

#### 2.B.1. Reference conditions for MV and kV beam parameters and reference scatterer

The reference conditions for the MV beam were a field size of 10.0 × 10.0 cm^2^, a dose rate of 400 MU/min, and gantry and ring angles of 0°. Those for the kV beam for each kV imaging subsystem were a kV collimator aperture size of 10.0 × 10.0 cm^2^ at the isocenter, a tube voltage of 110 kV, and exposure of 160 mA × 5 ms = 0.8 mAs/image.

The reference scatterer was a water‐equivalent cuboid phantom (Taisei Medical, Inc., Osaka, Japan; physical density: ~1 g/cm^3^; 30.0 × 30.0 × 26.0 cm^3^), which was placed at a source‐to‐surface distance of 90 cm. [Fig. 2(b)].

#### 2.B.2. MV‐scatter map acquisition with various MV beam parameters

Since both kV subsystems are always turned on during DTT treatment, the kV collimators were closed and two Pb plates (1 cm thickness) were deposited at the exits of each kV source to shield leaked kV x rays to acquire an image containing MV‐scatter only, that is, an MV‐scatter map. Ten orthogonal kV images were acquired by each kV imaging subsystem at a frame rate of 1 fps during MV beam irradiation. Those images were averaged, and the averaged image was used as the MV‐scatter map.

Each MV beam parameter was varied from its reference value while the other parameters were fixed to assess the dependency of each parameter, including the field size, dose rate, gantry angle, and ring angle. The details of the parametric variation are shown in Table 1. By defining a region of interest (ROI) of 100 × 100 pixels at the center of each MV‐scatter map, the pixel values in the ROI were averaged (MV‐scatter value). Thereafter, the MV‐scatter values were normalized to that obtained with the reference MV beam parameter values (a field size of 10.0 × 10.0 cm^2^, a dose rate of 400 MU/min, and gantry and ring angles of 0°). Herein, we call the relative MV‐scatter value the “MV‐scatter value factor.”

**Table 1:** Reference conditions for MV‐scatter map and variable parameters used in this study.

Parameter	Description
Reference conditions for MV‐scatter map acquisition	Field size: 10.0 × 10.0 cm^2^, dose rate: 400 MU/min, gantry and ring angles: 0°
X field size [cm]	2.0, 4.0, 6.0, 8.0, 10.0, 12.0, 14.0, 15.0
Y field size for each X field size [cm]	2.0, 4.0, 6.0, 8.0, 10.0, 12.0, 14.0, 15.0
Dose rate [MU/min]	100, 150, 200, 250, 300, 350, 400, 450, 500
Field size for each dose rate [cm^2^]	2.0 × 2.0, 4.0 × 4.0, 6.0 × 6.0, 8.0 × 8.0, 10.0 × 10.0, 12.0 × 12.0, 14.0 × 14.0, 15.0 × 15.0
Gantry angle [°]	0, 30, 60, 90, 120, 150, 180, 210, 240, 270, 300, 330
Ring angle [°]	−20, 0, +20

#### 2.B.3. kV‐scatter map acquisition with various kV beam parameters

To acquire an FPD1 (or FPD2) image containing kV‐scatter from kV Source 2 (or 1), the kV collimators of kV Source 1 (or 2) were closed and the Pb plate was deposited at the exit of kV Source 1 (or 2). Ten orthogonal kV images were acquired by each kV imaging subsystem at a frame rate of 1 fps without MV beam irradiation. The images from FPD1 (or FPD2) were averaged, and the averaged image was used as the kV‐scatter map for FPD1 (or FPD2). As in the procedure for the MV‐scatter maps, an ROI of 100 × 100 pixels at the center of each kV‐scatter map was defined, and the pixel values in that ROI were averaged (kV‐scatter value). Thereafter, the kV‐scatter values were normalized to that obtained with the reference MV beam parameters (a field size of 10.0 × 10.0 cm^2^, a dose rate of 400 MU/min, and gantry and ring angles of 0°). Herein, we refer to the relative kV‐scatter value as the “kV‐scatter value factor.”

Each kV beam parameter was varied from its reference value while the other parameters were fixed to assess the dependency of each parameter, including the tube voltage, exposure, kV collimator aperture size, gantry angle, and ring angle. The details of the parametric variations are shown in Table 2.

**Table 2:** Reference conditions for kV‐scatter map and variable parameters used in this study.

Parameter	Description
Reference conditions for kV‐scatter map acquisition	kV collimator aperture size: 10.0 × 10.0 cm^2^ at isocenter, tube voltage: 110 kV, exposure: 0.8 mAs/image, gantry angle: 0°
kV collimator aperture size [cm^2^ at isocenter]	10.0 × 10.0, 12.0 × 12.0, 14.0 × 14.0, 16.0 × 16.0, 22.0 × 17.0
Tube voltage [kV]	80, 90, 100, 110, 120, 125
exposure [mAs/image]	0.5, 0.8, 1.0, 1.25, 1.6
Gantry angle [°]	0, 30, 60, 90, 120, 150, 180, 210, 240, 270, 300, 330
Ring angle [°]	‐20, 0, +20

### 2.C. MV‐ and kV‐scatter correction experiment using intensity‐modulated beams

#### 2.C.1. Phantom setup and experimental procedure

To perform the MV‐ and kV‐scatter correction experiment, a phantom (QUASAR, Modus Medical Device, Inc., London, Canada) was used. A wooden rod (physical density: 0.4 g/cm^3^) with a 30‐mm‐diameter spherical pseudo‐tumor ball (target ball, physical density: 1.1 g/cm^3^) located at its center was employed, and two uniform acrylic rods were inserted into the sides of the wooden rod. The target ball in the wooden rod was surrounded peripherally by four gold markers (GMs; physical density: 19.3 g/cm^3^) whose centroid coincided with that of the target ball. The center of the target ball was positioned to coincide with the isocenter, and the longitudinal axis of the wooden rod was parallel to the superior–inferior axis. Figures 2(c) and 2(d) show the setup of the QUASAR phantom and a schematic cross‐section of the wooden rod.

Six intensity‐modulated MV beams were used for the experiment. The gantry and FPD angles for each beam are summarized in Table 3. The dose rate was 500 MU/min. The following kV beam parameters were used: a kV collimator aperture size of 10.0 × 10.0 cm^2^ at the isocenter, a tube voltage of 100 kV, and an exposure per image of 0.8 mAs. In the experiment, four types of images were obtained for each field: (a) images containing MV‐scatter only, (b) images containing kV‐scatter only, (c) concurrent kV images during MV beam irradiation (MV + kV images), and (4) kV images acquired without MV beam irradiation for reference (kV only images).

**Table 3:** Gantry angles, flat panel detector angles, and irradiated MUs of six intensity‐modulated megavoltage beams for the scatter correction experiment.

Beam #	Angle [°]			Irradiated MU
	Gantry	Flat panel detector 1	Flat panel detector 2	
1	160	25	295	217
2	120	345	255	202
3	80	305	215	308
4	280	145	55	256
5	240	105	15	364
6	200	65	335	272

#### 2.C.2. MV‐ and kV‐scatter correction

In the intensity‐modulated plan, MLC aperture size changes in each segment. Thereby, the images containing MV‐scatter only are obtained in each segment, and used as MV‐scatter map. kV‐scatter maps for each beam were generated as described in Sections 2.B.3, respectively. Each MV‐scatter map, kV‐scatter map, and combined MV‐ and kV‐scatter map was separately subtracted from the MV + kV images of each beam, and we call the subtracted images the MV‐scatter‐corrected (MVScorr), kV‐scatter‐corrected (kVScorr), and both MV‐ and kV‐scatter‐corrected (MVkVScorr) images. To evaluate the correction method, the intensity ratios of four GMs were calculated on each image and the kV‐only image. The intensity ratio was defined as *I_s_*/*I_m_*, where *I_m_* is the intensity of a pixel (*x*, *y*) at the center of the GM, and *I_s_* is the intensity of the pixel with the third lowest intensity obtained among the pixels located at positions (*x* ± 5, *y* ± 5), (*x* ± 5, *y* ∓ 5), (*x*, *y* ± 5), and (*x* ± 5, *y*).^16^ The intensity ratio was calculated using in‐house software developed in MATLAB 2018a (MathWorks, Natick, MA).

#### 2.C.3. Statistical analysis

To evaluate the difference between the kV only, MV + kV, MVScorr, kVScorr, and MVkVScorr images, the following statistical analyses were conducted. A test for equality of variance was performed among the images prior to multiple pairwise comparisons. According to the existence or nonexistence of equality of variance, a one‐way analysis of variance (ANOVA) test or the nonparametric Kruskal–Wallis test was performed to evaluate the differences between images. If the difference was significant, a nonparametric Steel–Dwass test was performed to evaluate the differences between images simultaneously. The level of significance for all tests was set at 0.05.

## 3 RESULTS

### 3.A. MV‐ and kV‐scatter maps

**Fig. 3:**
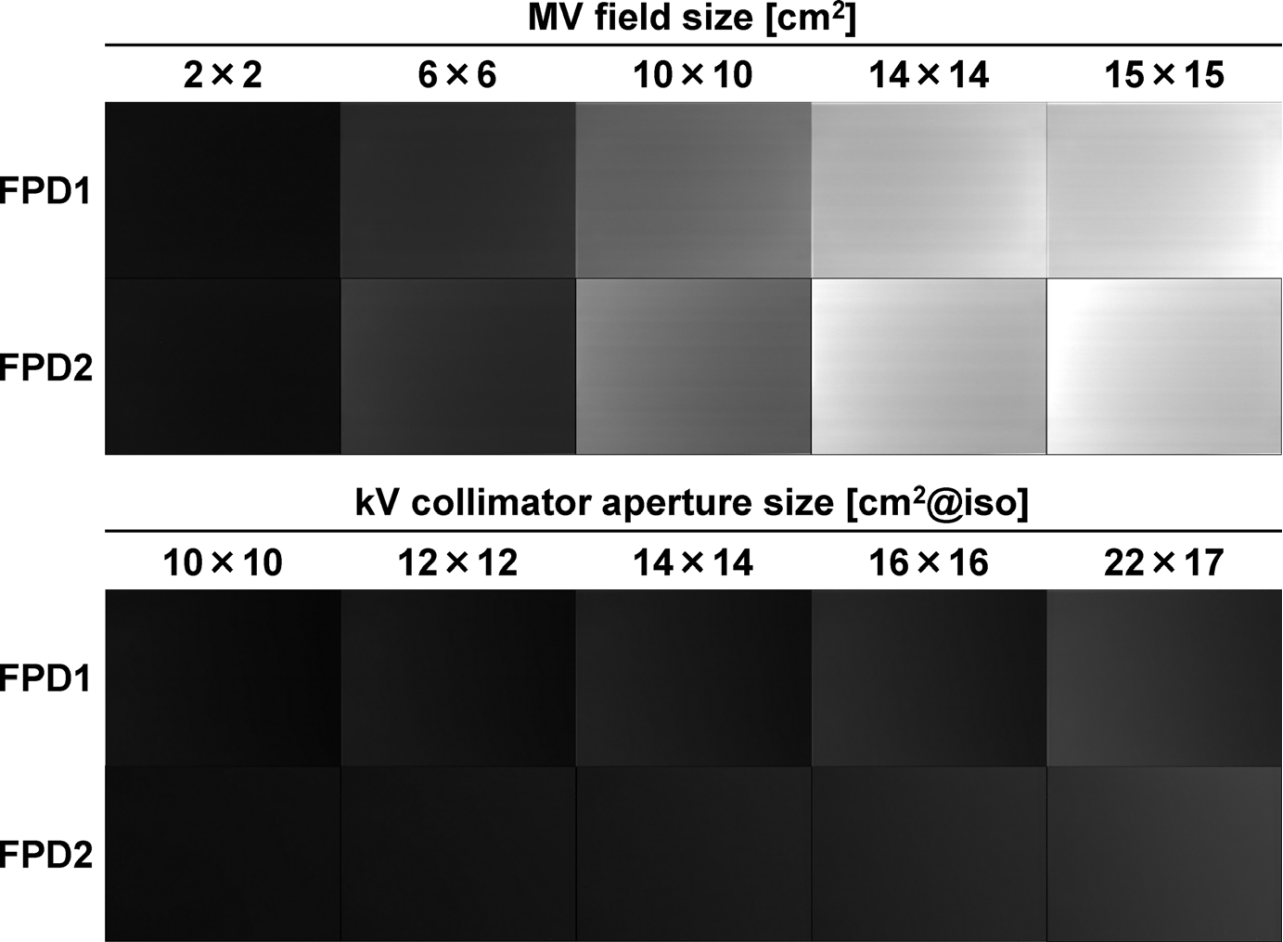
MV‐ and kV‐scatter maps for field sizes of 2.0 × 2.0, 6.0 × 6.0, 10.0 × 10.0, 14.0 × 14.0, and 15.0 × 15.0 cm^2^ and kV collimator aperture sizes of 10.0 × 10.0, 12.0 × 12.0, 14.0 × 14.0, 16.0 × 16.0, and 22.0 × 17.0 cm^2^ at the isocenter, respectively. The window levels and widths are 300 and 600.

**Fig. 4:**
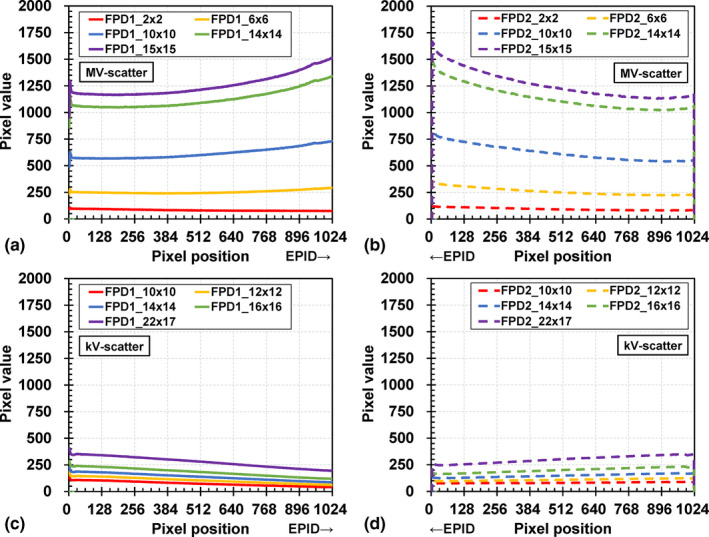
Pixel value profiles for the MV‐scatter maps acquired by (a) flat panel detectors1 (FPD1) and (b) FPD2, and those of kV‐scatter maps acquired by (c) FPD1 and (d) FPD2.

MV‐scatter maps for field sizes of 2.0 × 2.0, 6.0 × 6.0, 10.0 × 10.0, 14.0 × 14.0, and 15.0 × 15.0 cm^2^ and kV‐scatter maps for kV collimator aperture sizes of 10.0 × 10.0, 12.0 × 12.0, 14.0 × 14.0, 16.0 × 16.0, and 22.0 × 17.0 cm^2^ at the isocenter plane are shown in Fig. 3. The MV‐scatter qualitatively shows larger overall pixel values than the kV‐scatter does. For quantitative analysis, ROIs of 1024 × 100 pixels were defined at the center of each map [see the pixel value profiles in Figs. 4(a)–4(d)]. The amount of MV‐scatter is greater closer to the EPID.

### 3.B. Dependencies of MV beam parameters for MV‐scatter maps

#### 3.B.1. Field size dependency

**Fig. 5:**
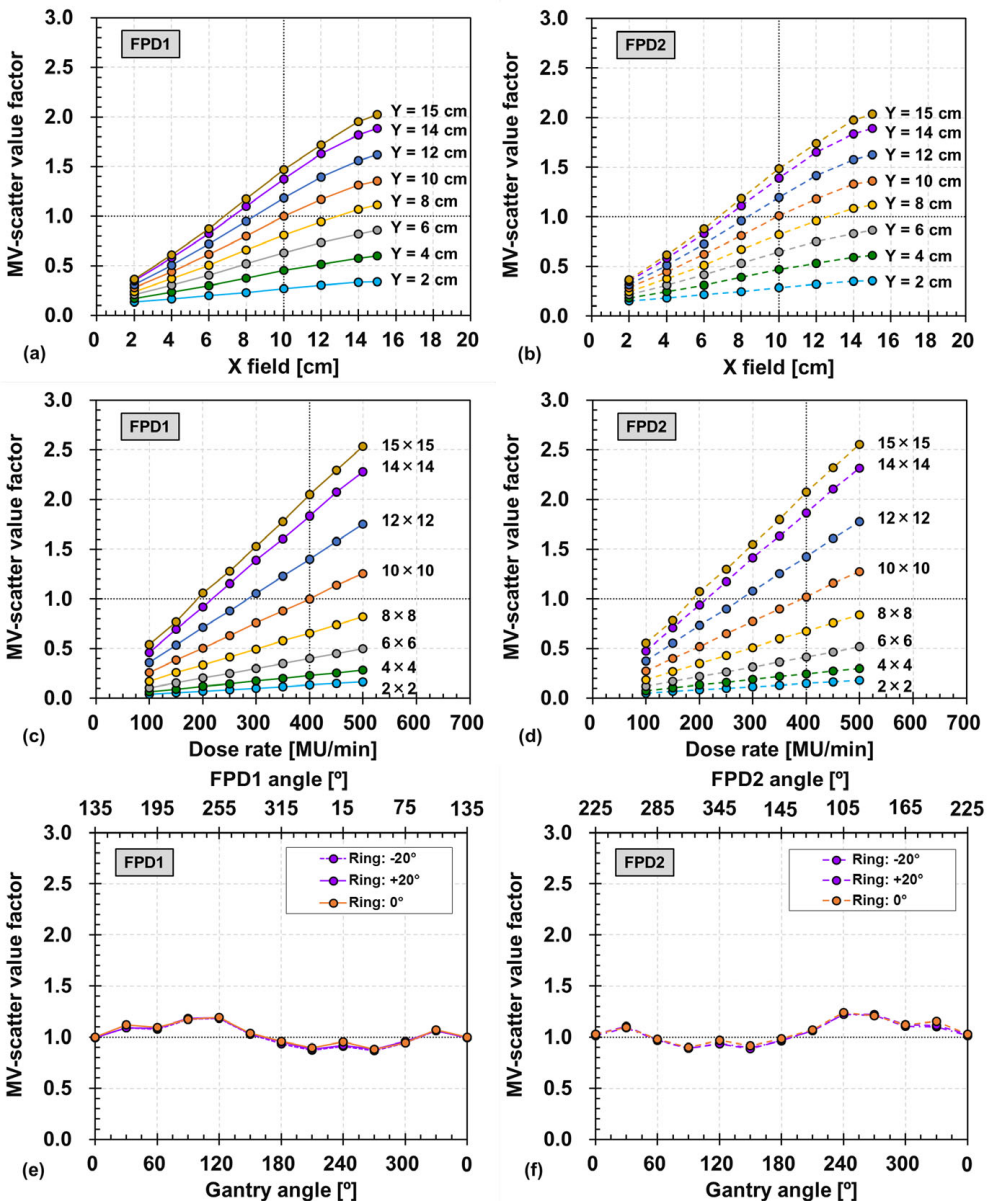
Field size dependencies of MV‐scatter acquired by (a) flat panel detectors1 (FPD1) and (b) FPD2. Dose rate dependencies of MV‐scatter acquired by (c) FPD1 and (d) FPD2. Gantry and ring angle dependencies of MV‐scatter acquired by (e) FPD1 and (f) FPD2.

The field size dependencies of FPD1 and FPD2 are shown in Figs. 5(a) and 5(b), respectively. The MV‐scatter value increases linearly with increasing field size. The maximum and minimum MV‐scatter value factors for FPD1 are 2.02 and 0.13 at field sizes of 15.0 × 15.0 and 2.0 × 2.0 cm^2^, respectively. The difference in MV‐scatter values between FPD1 and FPD2 with various field sizes is 0.00–0.02. This trend was observed for not only field size but also for other factors.

#### 3.B.2. Dose rate dependency

The dose rate dependencies with various field sizes for FPD1 and FPD2 are presented in Figs. 5(c) and 5(d), respectively. The MV‐scatter increases linearly with increasing dose rate since the number of MV photons increased and the number of MV‐scatters incident on each FPD increased with the same frame rate. The Pearson's coefficients of determination for each field size are 1, and the intercepts of the fitted lines are 0.

#### 3.B.3. Gantry and ring angle dependency

The gantry and ring angle dependencies of FPD1 and FPD2 are shown in Figs. 5(e) and 5(f), respectively. The maximum and minimum MV‐scatter value factors in FPD1 are 1.19 and 0.88 at a ring angle of 0° and gantry angles of 120° and 270° with FPD1 angles of 255° and 45°, respectively. The trend of FPD2 is symmetrical to that of FPD1 with respect to 180° rotation. The maximum difference between the MV‐scatter values at ring angles of 0° and ±20° is 0.05.

### 3.C. Dependencies of kV beam parameters for kV‐scatter maps

#### 3.C.1 kV collimator aperture size dependency

**Fig. 6:**
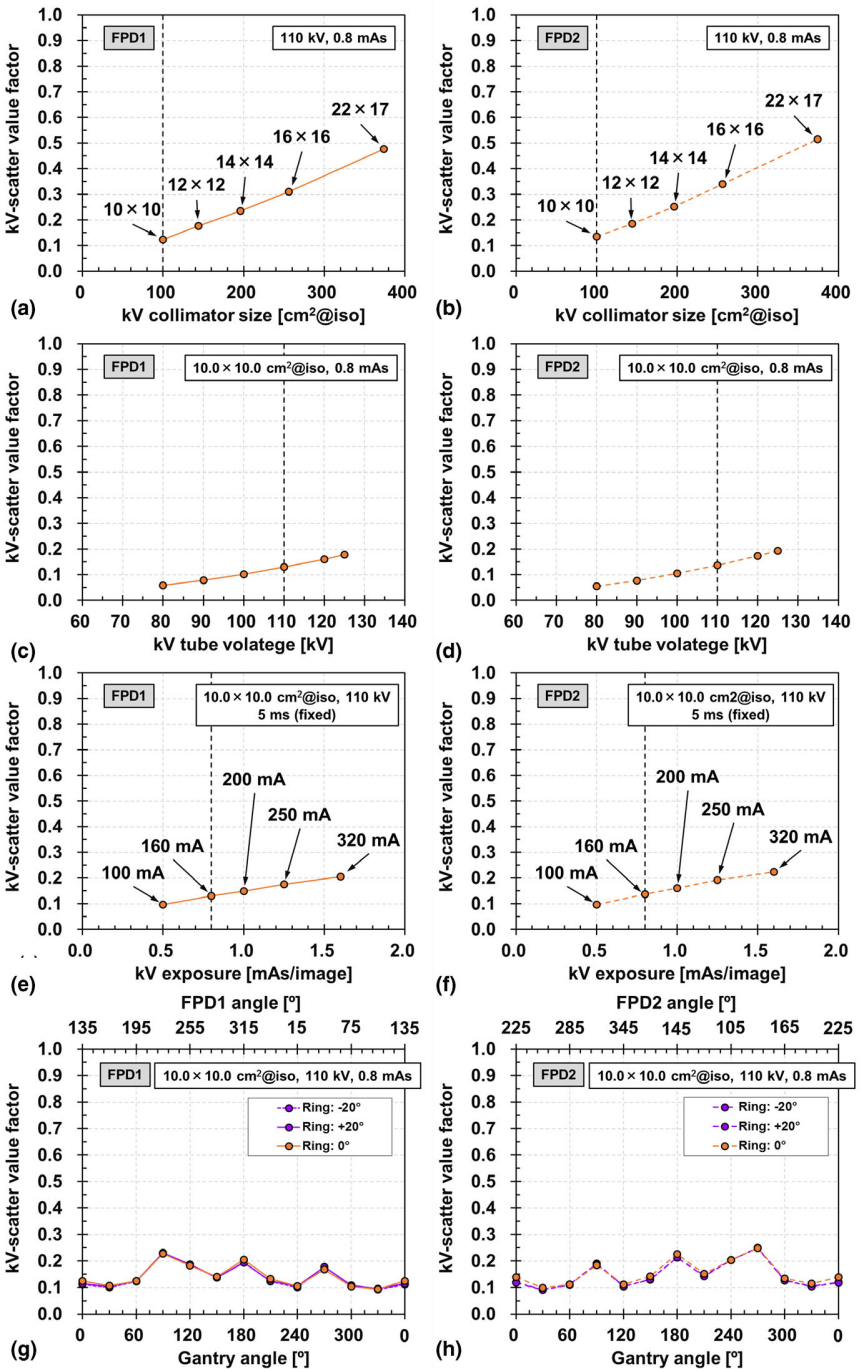
kV collimator aperture size dependencies of kV‐scatter acquired by (a) flat panel detectors1 (FPD1) and (b) FPD2. Tube voltage dependencies of kV‐scatter acquired by (c) FPD1 and (d) FPD2. Exposure dependencies of kV‐scatter acquired by (e) FPD1 and (f) FPD2. Gantry and ring angle dependencies of kV‐scatter acquired by (g) FPD1 and (h) FPD2.

The kV collimator aperture size dependencies for FPD1 and FPD2 are depicted in Figs. 6(a) and 6(b), respectively. The kV‐scatter value increases linearly with increasing aperture size. However, the kV‐scatter value is much smaller than the MV‐scatter value. For instance, the maximum kV‐scatter value factor for FPD1 is 0.48, which occurs at the fully open aperture size of 22.0 × 17.0 cm^2^. The difference in kV‐scatter values between FPD1 and FPD2 with various field sizes is 0.00–0.04. This trend was observed for both the field size and other factors.

#### 3.C.2. Tube voltage and exposure dependency

The tube voltage dependencies of FPD1 and FPD2 are presented in Figs. 6(c) and 6(d), respectively, and their exposure dependencies are shown in Figs. 6(e) and 6(f), respectively. The kV‐scatter value increases linearly with increasing tube voltage and exposure. However, the maximum kV‐scatter value factors for FPD1 are 0.18 and 0.21, which occur at a tube voltage of 125 kV and an exposure of 1.6 mAs, respectively.

#### 3.C.3. Gantry and ring angle dependency

The gantry and ring angle dependencies of FPD1 and FPD2 are shown in Figs. 6(g) and 6(h), respectively. As in the MV‐scatter case, the trend of FPD2 is symmetrical to that of FPD1 with respect to 180° rotation. The maximum difference between the MV‐scatter values at ring angles of 0° and ±20° is 0.07.

### 3.D. MV‐ and kV‐scatter correction experiment using intensity‐modulated beams

**Fig. 7:**
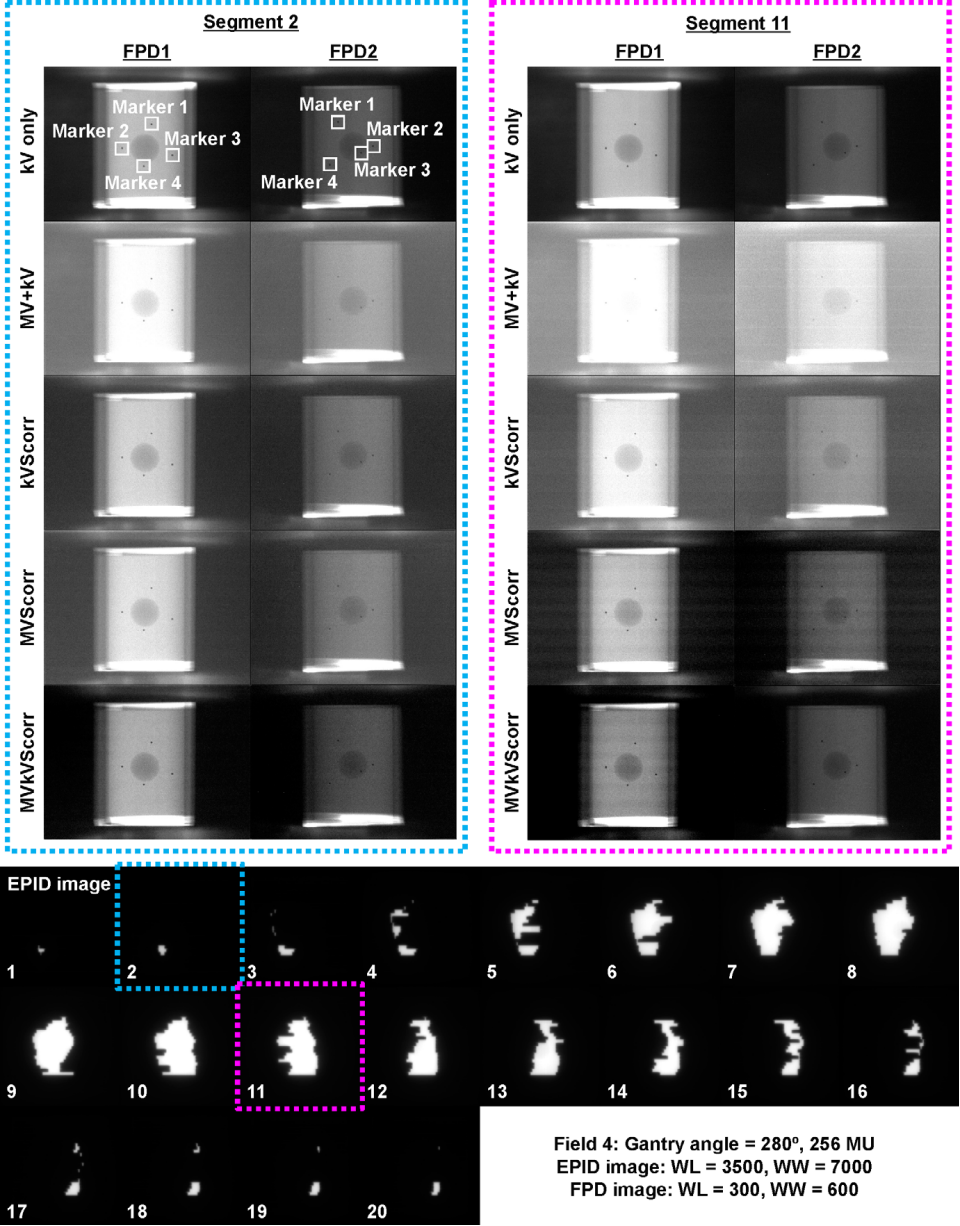
Reference kV image without MV beam irradiation (kV only), concurrent kV image during MV beam irradiation (MV + kV), kV‐scatter‐corrected image (kVScorr), MV‐scatter‐corrected image (MVScorr), MV‐ and kV‐scatter‐corrected image (MVkVScorr) for flat panel detectors1 (FPD1) and FPD2 obtained at segments 2 and 11, and EPID images of Field 4. x‐ray path of FPD2 was longer than that of FPD1. The window levels and widths for EPID images are 3500 and 7000. The window levels and widths for FPD images are 300 and 600.

**Fig. 8:**
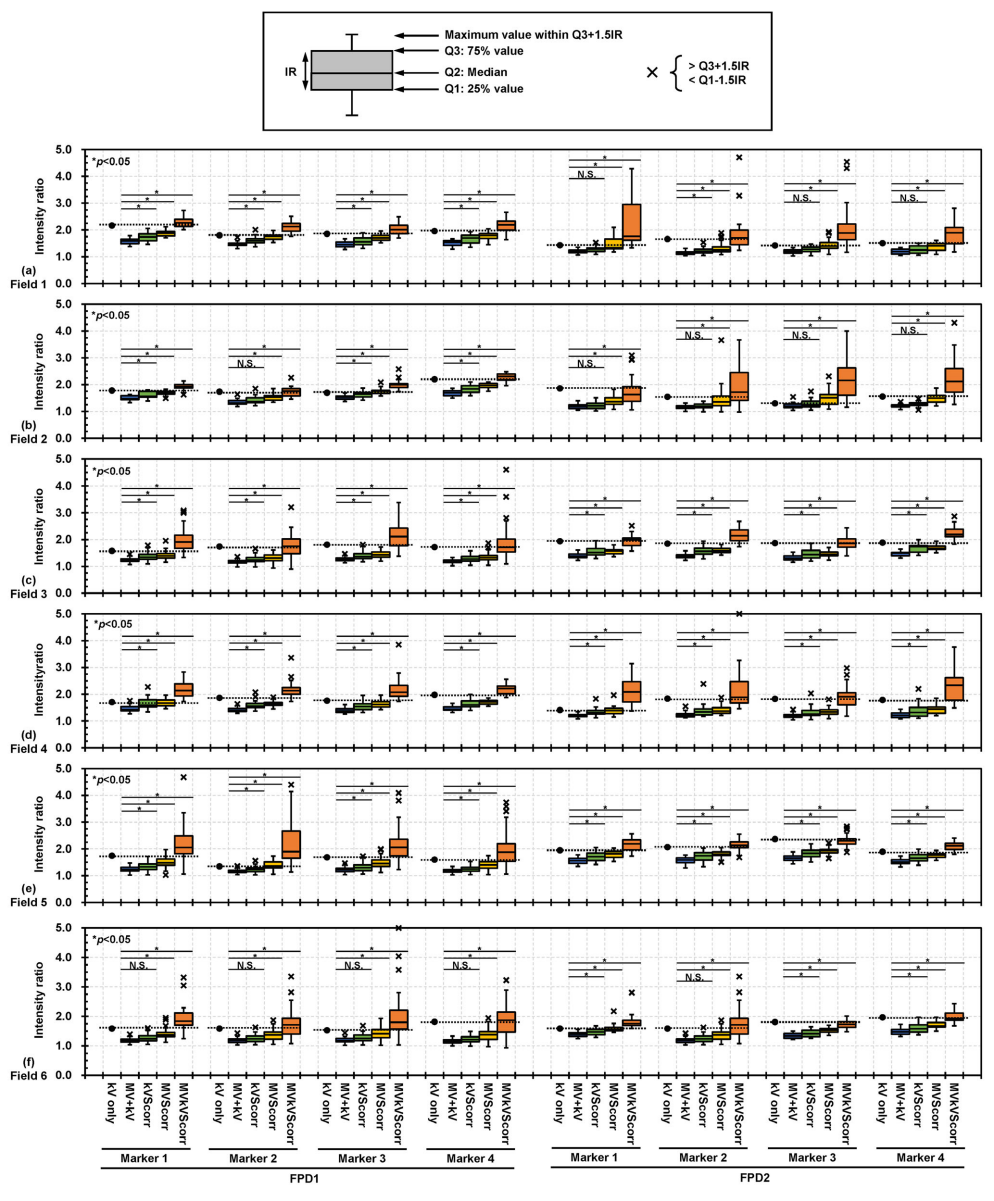
Boxplots of intensity ratios of gold markers (GMs) on reference kV images without MV beam irradiation (kV only) and in concurrent kV images acquired during MV beam irradiation (MV + kV), kV‐scatter‐corrected images (kVScorr), MV‐scatter‐corrected images (MVScorr), and MV‐ and kV‐scatter‐corrected images (MVkVScorr) acquired by flat panel detectors1 (FPD1) and FPD2 at (a) Field 1, (b) Field 2, (c) Field 3, (d) Field 4, (e) Field 5, and (f) Field 6. *P < 0.05, N.S.: not significant.

The kV only, MV + kV, kVScorr, MVScorr, and MVkVScorr images for FPD1 and FPD2 obtained at segments 2 and 11, as well as EPID images of Field 4 are presented in Fig. 7. Both the MV‐ and kV‐scatter were corrected in the MVkVScorr images, which look similar to the corresponding kV‐only images. However, stripe bands are observable in the MV + kV images, which could not be eliminated by MV‐ or kV‐scatter map subtraction, as they were caused by electric noise. Boxplots of the intensity ratios of the kV only, MV + kV, kVScorr, MVScorr, and MVkVScorr images for FPD1 and FPD2 obtained at each field size are shown in Fig. 8. For all markers and any field, the intensity ratios in the MV + kV images are decreased compared to the reference ones in terms of both MV‐ and kV‐scatter. The x‐ray scatter corrections gradually improved the intensity ratios in the kVScorr, MVScorr, and MVkVScorr images. The intensity ratios of all of the scatter‐corrected images are significantly larger than those of the uncorrected MV + kV images, except for several kVScorr images (*P* < 0.05). In particular, the intensity ratios of the MVkVScorr images are comparable to those of the kV‐only images.

## 4 DISCUSSION

To the best of our knowledge, this is the first study in which x‐ray scatter on the orthogonal kV imaging subsystems of Vero4DRT has been measured directly. MV‐scatter and kV‐scatter from the other kV source were included in the kV projections during DTT treatment and degraded the image quality. MV‐ and kV‐scatter maps were obtained and quantified under various MV and kV beam parameters, respectively. In addition, an MV‐ and kV‐scatter correction experiment was performed using a phantom under intensity‐modulated beam irradiation.

The pixel value profiles of the MV‐scatter maps in FPD1 and FPD2 in Figs. 4(a) and 4(b), respectively, revealed the MV‐scatter distribution clearly. MV‐scatter was distributed toward the side close to the EPID or MV beam, as the forward‐scattered x rays were dominant in the MV beam region. This finding is supported by the Klein–Nishina formula.^19^ More MV‐scatter was incident on the FPD with increasing field size, which is supported by Figs. 5(a) and 5(b). The field size was one of the main factors determining the MV‐scatter, and the dose rate was the dominant parameter. As shown in Figs. 5(c) and 5(d), the MV‐scatter increased linearly with increasing dose rate, because the dose rate is equivalent to the number of x rays per minute. In this study, the kV‐scatter values were normalized to the MV‐scatter value obtained under the reference MV beam conditions. Thus, the kV‐scatter values were smaller than the MV‐scatter values. The kV‐scatter value factor was 0.12 under the reference kV beam conditions. The dominant kV beam parameter in determining the kV‐scatter was the kV collimator aperture size.

For the MV‐ and kV‐scatter correction experiment, a clinical treatment scenario was assumed. As shown in Fig. 7, a larger field size (segment 11) yielded more MV‐scatter in the MV + kV images, as supported by the direct measurements (Fig. 5). In addition, not only MV‐scatter correction but also kV‐scatter correction is necessary since the intensity ratio was improved in the kVScorr images, even though the amount of kV‐scatter was less than that of MV‐scatter.

According to the obtained results, MV‐ and kV‐scatter have greater effects on monitoring images during DTT treatment when the field and kV collimator aperture are large and the dose rate is high. The monitoring images of a patient who has a large target or an implanted marker movement are potentially affected by MV‐ and kV‐scatter, as the field size or kV collimator aperture size may be large. Thus, care must be taken in DTT treatment, particularly that for pancreatic cancer, because the field size and kV collimator aperture size in pancreatic cancer treatment are larger than those in lung or liver cancer treatment.

Degradation of the intensity ratio in concurrent orthogonal kV images for monitoring in terms of both MV‐ and kV‐scatter would cause failure of the automatic beam‐off system, which is based on the detected radiopaque marker positions in the monitoring images. In case the marker positions cannot be detected in the monitoring image, the automatic beam‐off system is turned off, and an operator of the Vero4DRT system then interrupts the MV beam manually if the predicted marker position is out of the tolerance range by assessing the displayed monitoring images. In addition, online rebuilding of the 4D model cannot be employed if the marker positions in the monitoring images are not available. Additional kV imaging is necessary to rebuild the 4D model. Thereby, the manual beam‐off and offline rebuilding of the 4D model may cause unnecessary additional kV imaging exposure to patients. The mean imaging doses to bone caused by monitoring and building the 4D model during lung DTT treatment are approximately 3.0 and 5.0 cGy, respectively.^20^

To improve the image quality of monitoring images acquired during DTT treatment, online MV‐ and kV‐scatter correction is necessary. In clinical practice, MV‐ and kV‐scatter maps should be generated for each patient. To obtain such patient‐specific scatter maps, Monte Carlo (MC) simulation is one method that does not cause additional patient exposure. In this approach, patient‐specific scatter maps can be simulated by inputting the planning CT data and plan into the dedicated MC geometry. The obtained data in this study can be used to validate the dedicated MC geometry.

One of the limitations of this study is that only a 6 MV beam was used, while recent linear accelerators (LINACs) have employed other MV beam energies. According to the Klein–Nishina formula, the proportion of side‐scattering decreases with increasing MV beam energy.^19^ Thus, MV beam energies >6 MV would have lower MV‐scatter values. In addition, the adaptability of this study is limited because of the FPD positions chosen. LINACs provided by other vendors, such as Varian or Elekta, have only single kV imaging subsystems mounted perpendicular to the MV beam. Although the CyberKnife system has two kV imaging subsystems, they are fixed, and the linear accelerator is mounted on a robotic arm.

## 5 CONCLUSION

This is the first study in which both MV‐ and kV‐scatter on two orthogonal kV imaging subsystems mounted on the Vero4DRT system have been measured. The MV‐scatter increased with increasing field size and dose rate, and the kV‐scatter increased with increasing kV collimator aperture size and exposure. The gantry and ring angles had little effect on either type of scatter. The data provided by this study can serve as fundamental evidence regarding MV‐ and kV‐scatter correction in DTT treatment using Vero4DRT. In addition, our MV‐ and kV‐scatter correction experiment with intensity‐modulated beams showed improvement of the intensity ratio calculation for the GMs. To facilitate more accurate DTT treatment, both MV‐ and kV‐scatter values should be corrected online.

## CONFLICT OF INTEREST

The authors of this publication have no conflict of interest to declare.
